# Treatment of Hay Fever

**Published:** 1883-09

**Authors:** 


THE TREATMENT OF HAY-FEVER.—Mr. W. F. Phillips, of St. Mary Bourne, Andover, writes: It is just over five weeks since a lady placed herself under my care for the treatmentof hay-fever, or summer catarrh—a very much better name. She had suffered severely for many years, and sometimes from the end of May to near the end of July, with little or no intermission, unless she kept indoors. Her mother, it is worthy of remark, was very sensitive to the odor of certain flowers, and was affected by some of them even to the extent of fainting. She was not subject, however, to summer catarrh.

Knowing how exceedingly unsatisfactory is the treatment recommended and practiced for this disease, as is sufficiently evident from the recent communications to the *Journal* on the subject, I sought for rational indications that might guide me to the selection of a remedy. I thought of the neurosis that seems to underlie most cases of this kind, and to constitute the essential cause or predisposition on which the disease depends; of the characteristic symptoms of the malady; the injection of the conjunctiva, the hyperæmia and hyperæsthesia of the nasal cavities, the excessive secretion of tears and mucus, and then I bethought me of a drug whose physiological action might indicate the possession of the power to control such symptoms. Belladonna was the drug that suggested itself at once, and I determined to give it a trial, all the more hopefully because I remembered how strikingly useful on similar indications, and by a parity of reasoning, I had often found it in ordinary conjunctivitis and simple catarrh. I began with the following prescription: R Succi belladonnæ *m*xxiv; aquam ad ℥iij. Misce. A teaspoonful to be taken every hour till relief is obtained. The medicine was taken without the production of any undesirable effect, and with very marked advantage indeed—an advantage that became still more evident and unmistakable, both to the patient and myself, when the dose was increased from one minim to one and a quarter (half a drachm in three ounces). Once, too, when the eyelids were especially tender, the patient was advised to use the mixture as a lotion to the affected parts, and this local application was found to be a most useful addition to the internal administration of the remedy. Repeatedly, when the symptoms of an attack had been allowed to begin, the patient found prompt relief after a few doses of the drug, the catarrhal affection disappearing first, and then the asthmatic; and on taking it regularly every day after the malady had been subdued, she has found to her delight that she can take her walks abroad through blooming grass and flowers without the least protection or precaution—a thing she had not been able to do before for years.

The patient, remembering no doubt the failure of past treatment, pronounces the remedy "a great success;" but, however satisfactory the case may be, it is, as far as I know, a solitary one, and therefore stands in need of confirmation and support.—*British Medical Journal.*

